# Immunodominant antibody responses directed to SARS-CoV-2 hotspot mutation sites and risk of immune escape

**DOI:** 10.3389/fimmu.2022.1010105

**Published:** 2023-01-05

**Authors:** Jamille Ramos Oliveira, Cesar Manuel Remuzgo Ruiz, Rafael Rahal Guaragna Machado, Jhosiene Yukari Magawa, Isabela Pazotti Daher, Alysson Henrique Urbanski, Gabriela Justamante Händel Schmitz, Helen Andrade Arcuri, Marcelo Alves Ferreira, Greyce Luri Sasahara, Giuliana Xavier de Medeiros, Roberto Carlos Vieira Silva Júnior, Edison Luiz Durigon, Silvia Beatriz Boscardin, Daniela Santoro Rosa, Deborah Schechtman, Helder I. Nakaya, Edecio Cunha-Neto, Gabriele Gadermaier, Jorge Kalil, Verônica Coelho, Keity Souza Santos

**Affiliations:** ^1^ Departamento de Clínica Médica, Disciplina de Alergia e Imunologia Clínica, Faculdade de Medicina da Universidade de São Paulo, São Paulo, SP, Brazil; ^2^ Laboratório de Imunologia, LIM19, Instituto do Coração (InCor), Hospital das Clínicas da Faculdade de Medicina da Universidade de São Paulo, (HCFMUSP) São Paulo da Universidade de São Paulo, São Paulo, Brazil; ^3^ Instituto de Investigação em Imunologia–Instituto Nacional de Ciências e Tecnologia – instituto de investigação em imunologia - Instituto Nacional de Ciências e Tecnologia (iii-INCT), São Paulo, Brazil; ^4^ Departamento de Microbiologia, Instituto de Ciências Biomédicas, Universidade de São Paulo, São Paulo, SP, Brazil; ^5^ Departamento de Análises Clínicas e Toxicológicas, Faculdade de Ciências Farmacêuticas, Universidade de São Paulo, São Paulo, SP, Brazil; ^6^ Centro de Estudos de Insetos Sociais, Departamento de Biologia, Instituto de Biociências de Rio Claro, Universidade Estadual Paulista, Rio Claro, SP, Brazil; ^7^ Laboratório de Biologia Celular, Laboratório de Investigação Médica 59 (LIM59), Departamento de Patologia, Faculdade de Medicina Faculdade de Medicina da Universidade de São Paulo (FMUSP), Universidade de São Paulo, São Paulo, Brazil; ^8^ Plataforma Científica Pasteur-USP, São Paulo, SP, Brazil; ^9^ Departamento de Parasitologia, Instituto de Ciências Biomédicas, Universidade de São Paulo, São Paulo, SP, Brazil; ^10^ Departamento de Microbiologia, Imunologia e Parasitologia, Universidade Federal de São Paulo (UNIFESP/EPM, São Paulo, SP, Brazil; ^11^ Departamento de Bioquímica, instituto de Química, Universidade de São Paulo, São Paulo, SP, Brazil; ^12^ Hospital Israelita Albert Einstein, São Paulo, SP, Brazil; ^13^ Department of Biosciences and Medical Biology, Paris Lodron University Salzburg, Salzburg, Brazil

**Keywords:** linear antibody epitopes, peptide array, RBD, immune pressure, sarbecovirus, betacoronavirus

## Abstract

**Introduction:**

Considering the likely need for the development of novel effective vaccines adapted to emerging relevant CoV-2 variants, the increasing knowledge of epitope recognition profile among convalescents and afterwards vaccinated with identification of immunodominant regions may provide important information.

**Methods:**

We used an RBD peptide microarray to identify IgG and IgA binding regions in serum of 71 COVID-19 convalescents and 18 vaccinated individuals.

**Results:**

We found a set of immunodominant RBD antibody epitopes, each recognized by more than 30% of the tested cohort, that differ among the two different groups and are within conserved regions among betacoronavirus. Of those, only one peptide, P44 (S415-429), recognized by 68% of convalescents, presented IgG and IgA antibody reactivity that positively correlated with nAb titers, suggesting that this is a relevant RBD region and a potential target of IgG/IgA neutralizing activity.

**Discussion:**

This peptide is localized within the area of contact with ACE-2 and harbors the mutation hotspot site K417 present in gamma (K417T), beta (K417N), and omicron (K417N) variants of concern. The epitope profile of vaccinated individuals differed from convalescents, with a more diverse repertoire of immunodominant peptides, recognized by more than 30% of the cohort. Noteworthy, immunodominant regions of recognition by vaccinated coincide with mutation sites at Omicron BA.1, an important variant emerging after massive vaccination. Together, our data show that immune pressure induced by dominant antibody responses may favor hotspot mutation sites and the selection of variants capable of evading humoral response.

## Introduction

SARS-CoV-2 vaccines have been mainly evaluated based on the generation of B cell responses to induce the production of neutralizing antibodies. The receptor binding domain (RBD) in the S1 subunit of the SARS-CoV-2 spike glycoprotein is responsible for binding to the aminopeptidase N region of the angiotensin converting enzyme-2 receptor (ACE-2) and is the main binding receptor for the entry of SARS-CoV-2 into human cells (1, 2), making it a relevant target for the development of neutralizing antibodies, inhibitors, and vaccines (2, 3). Serological studies performed with a cohort of 647 SARS-CoV-2-infected individuals have shown that RBD is immunodominant within the spike protein and targeted by 90% of the neutralizing activity of circulating antibodies (4).

Despite the vast amount of data on SARS-CoV-2 infection, generated since the 2019 pandemic outbreak, many important issues remain poorly understood. So far, RBD has been reported to essentially lack sequential epitopes for antibody binding (3, 5, 6). A deeper understanding of SARS-CoV-2 antibody responses directed to different epitopes throughout the course of infection, their potential involvement in different disease outcomes, and the identification of immunodominant epitopes await deeper investigation. It is not clear if antibody responses directed to multiple or to some dominant SARS- CoV-2 antigenic regions account for significant neutralizing capacity and whether responses to specific epitopes are associated with distinct clinical outcomes.

Some concern has emerged regarding the rapid evolution (7, 8) of the virus, with a concomitant decrease or loss of neutralization activity against novel variants (9–11). Exploring the RBD antibody epitope profile can allow the identification of immunodominant regions and mutation hotspots, among different variants, providing information to explore immune pressure, selection, surges of new variants, critical to improve vaccine strategies to face emerging variants of SARS- CoV-2.

We analyzed the linear epitope landscape of SARS-CoV-2 RBD IgG and IgA antibody responses, to identify potential immunodominant epitopes among convalescents from the first wave of infection. Eight immunodominant epitopes were detected. The top one contains a hotspot mutation site (K417) present in all VOCs before omicron surge and presents intensity of antibody reactivity directly correlated with neutralizing titers. The epitope profile recognition following vaccination differed from convalescents and displayed immunodominant peptides within regions that are mutated in omicron.

## Materials and methods

### Study population

Seventy-one COVID-19 convalescent individuals (52% female, 48% male, median age 42 years old) with diagnostic confirmation by RT-PCR from March to April 2020 were included in this study. Samples were collected 30-50 days after symptoms onset at the *Hospital das Clínicas da Universidade de São Paulo*, Brazil. Participants were selected from a larger cohort and grouped based on displaying high (≥1:160) or low (<1:160) neutralization titers. Of the selected individuals, 21 individuals had been hospitalized, of which 9 were in the intensive care unit without mechanical ventilation. Fifty participants (70%) presented mild symptoms that did not require hospitalization. Eighteen vaccinated individuals (77,78% female, 22,22% male, median age 52,5 years old) were selected from whom two samples were collected: one 15 days after second dose of Coronavac and the other 90 days after a booster with miRNA-273 Pfizer vaccine. The study was approved by CAPPesq (*Comissão de Ética Para Análise de Projetos de Pesquisa do HC-FMUSP*) and CONEP (*Comissão Nacional de Ética em Pesquisa) (CAAE: 30155220.3.0000.0068*). All study participants signed informed consents.

### Serum IgG ELISA specific for RBD

Ninety-six-well high-binding half-area polystyrene plates (Corning, USA) were coated with 25 µL of 1 mg/mL RBD protein (GenScript Biotech, USA) diluted with carbonate-bicarbonate buffer (pH 9.6, 0.1 M) and incubated overnight at 4°C. Coat solutions were discarded and 80 µL of 1% BSA, 5% non-fat dried milk and 0.05% Tween-20 in phosphate saline buffer (PBS) was added to each well to block. Blocking was performed at room temperature for 2 h. Serum or plasma samples were thawed at room temperature and incubated at 56°C for 30 min for inactivation. The samples were then diluted in 0.25% BSA, 5% non-fat dried milk and 0.05% Tween-20 in PBS. Blocking solution was discarded and 50 µL of serum solutions diluted at 1:100 was added to each well and the plates were incubated at 37°C for 45 min. Following the incubation step, plates were washed five times with 0.05% Tween-20 in PBS. Secondary antibody solution of goat anti-human IgG conjugated to peroxidase (Jackson Immunoresearch, USA) diluted 1:10,000 in 0.25% BSA, 5% non-fat dried milk and 0.05% Tween-20 in PBS was prepared and 25 µL added to each well. Plates were incubated at 37°C for 30 min and then washed five times. o-Phenylenediamine dihydrochloride (OPD) tablets (Sigma, USA) were dissolved in 0.05 M phosphate-citrate buffer (pH 5.0) at a concentration of 0.4 mg/mL. Immediately prior to use, 5 µL of 30% hydrogen peroxide was added to the solution. Then, 50 µL of the final solution was added to each well and the plates were incubated in the dark at room temperature for 30 minutes. After the incubation period, the reaction was stopped by adding 50 µL of 2N H_2_SO_4_ solution to each well. The plates were then read at 490 nm on a plate reader (GloMax, Promega, USA).

Results are given as the ratio of the individual sample/control sample. An antibody ratio ≥1.2 was considered positive.

### RBD peptide array

The mapping of IgG and IgA-specific epitopes was performed by microarray using PEPperMAP^®^ Linear Epitope Mapping from PEPperPRINT (Heidelberg, Germany). The SARS-CoV-2 Spike RBD sequence (S_335_ to S_516_) was synthesized as overlapping peptides of 15 amino acid residues in length with 13 overlapping residues, totalizing 91 peptides (**Supplementary Table 1**). Peptides were printed in duplicates onto glass slides. Each chip contained peptides derived from Influenza Hemagglutinin and Polio Virus as positive controls.

To ensure that the secondary antibodies do not unspecifically interact with the antigen-derived peptides printed on the arrays, a copy of the array was pre-stained with goat anti-human IgG (H+L) DyLight680 (Invitrogen, USA) secondary antibody or goat anti-human IgA (chain alpha) DyLight800 (Rockland Immunochemicals Inc., USA) diluted 1:2000 in staining buffer (PBS with 10% blocking buffer) and incubated at room temperature for 45 min on an orbital shaker. No background fluorescence due to nonspecific binding of the secondary antibody was observed. Subsequently, serum samples from convalescent individuals were serially diluted from 1:10 to 1:1000 in staining buffer. The best dilution of 1:10 was chosen and added to the microarrays for overnight incubation at 4°C. After three washing steps of 1 min each with 200 µL of the standard buffer, microarrays were incubated with anti-IgG and anti-IgA on an orbital shaker at room temperature for 45 minutes. Following secondary antibody incubation, three wash steps were performed and microarrays were dipped in dipping buffer (1 mM TRIS, pH 7.4) and centrifuged at 250 g for 5 minutes for drying.

### Peptide microarray spot quantification

Fluorescence signals on microarrays were detected with an Odyssey Scanner (LI-COR Biosciences, USA). The quantification of spot intensities and peptide annotation were performed using GenePix Pro 4.0 (Molecular Devices, USA). The software analysis provided fluorescence intensities (FI) broken down into raw, foreground, and background signal. Mean fluorescence was calculated subtracting background from raw values. The foreground mean FI of reactivity to each peptide was averaged over the duplicates, and signal-to-noise ratios were additionally calculated for each peptide spot. Duplicate mean values for each peptide were plotted as a heat map of the median value from the 91 spots separately for IgG and IgA reactivity. The heatmap was generated using the pheatmap R package (v1.0.12). For the heatmap, MFI (mean fluorescence intensity) values were standardized by adding +1 and then applying log2.

### Virus neutralization assay

SARS‐CoV‐2 (GenBank: MT MT350282) was used to conduct a cytopathic effect (CPE)‐based virus neutralization test (VNT), as previously described (12). We used 96‐well plates containing Vero cells (ATCC CCL‐81), at 5 x 10^4^ cells/mL. Inactivated sera were diluted (1:20 to 1:5120) for the assay. Serum dilutions were mixed in equal volumes with the virus (100 tissue culture infectious doses, 100% endpoint per well – VNT_100_) and pre-incubated for virus neutralization for 1 hour at 37°C. The mixtures containing serum and virus were transferred to the confluent cell monolayer and incubated at 5% CO_2_ for 3 days at 37°C. After 72 hours, plates were analyzed by light microscopy. Gross CPE was observed on Vero cells, distinguishing the presence or absence of CPE‐VNT. To determine the neutralizing antibody titers, the highest serum dilution that was able to neutralize virus growth was considered. This was confirmed by fixing and staining plates with amido black (0.1% amido black [w/v] solution with 5.4% acetic acid, 0.7% sodium acetate) for 30 min. As a positive control, an internal serum from a RT‐qPCR positive individual with a plaque reduction in the neutralization test >1:640 was used in each assay. Following recommendations of the World Health Organization, all cytopathic effect-based virus neutralization assays were performed in a Biosafety Level 3 laboratory. The study individuals were clustered into two groups, resulting in 52 with high neutralization capacity (≥1:160 titers) and 19 individuals with low neutralization (<1:160 titers) (**Supplementary Figure S1A**), following the EU recommendation for COVID-19 plasma donation (13). Neutralizing antibody titers were transformed into natural logarithms (ln) for normal distribution.

### *In silico* B cell epitope prediction

Linear B cell epitopes of RBD were predicted using the BepiPred-2.0 web server (http://www.cbs.dtu.dk/services/BepiPred/) (14). BepiPred-2.0 is based on a random forest algorithm trained on epitopes annotated from antibody-antigen protein structures. This method was found to be superior to other available tools for sequence-based epitope prediction, with regard to epitope data derived from solved 3D structures and a large collection of linear epitopes downloaded from the IEDB database, respectively. In this study, we used a threshold value of 0.55.

### Comparative amino acid sequence analysis

Receptor-binding domain (RBD) sequence of spike protein from SARS-CoV-2 (319-540, Genbank: QIG55955) and other sarbecoviruses such as SARS-CoV (306-526, Genbank: AAR86775), Civet-SARS SZ3 (306-526, Genbank: AAU04646), Pangolin-CoV (319-540, Genbank: QIQ54048), Bat-CoV Rs3367 (307-527, Genbank: AGZ48818) and Bat-CoV RaTG13 (319-540, Genbank: QHR63300) were aligned using the MUSCLE program (https://www.ebi.ac.uk/Tools/msa/muscle/) (15). Results were exported to Weblogo website (http://weblogo.threeplusone.com) (16) to generate a graphical representation of the multiple sequences alignment.

### Structural analyses

Structural representations of RBD interacting with ACE2 (pdb: 6M0J) and with the neutralizing antibodies B38 (pdb: 7BZ5) and COVA1-16 (pdb: 7JMW) were generated using UCSF Chimera (https://www.cgl.ucsf.edu/chimera/) (17).

### Statistical analyses

GraphPad Prism 9.0.1 was used for statistical analyses of individual peptide reactivity comparing sera from individuals with high and low neutralization capacity (Mann-Whitney), Spearman for correlation analysis, and p values <0.05 were considered statistically significant. The one-sided Mann-Whitney test was performed to investigate differences between the mean MFI values of high and low neutralization groups, for each peptide, using the rstatix R package (v0.6.0). To visualize results, -log2 (p-values) were plotted in a heatmap using the ggpubr package (0.4.0). The statistical power (1-β) of 50 (VNT ≥1:160) versus 21 (VNT <1:160) individuals for *P44* was calculated.

## Results

### IgG and IgA peptide recognition profile

Individuals were grouped into two groups: (i) 52 individuals with high neutralization (≥1:160 titers), (ii) 19 individuals with low neutralization (<1:160 titers). Among the low neutralizers, the majority presented 1:40 titers, while among the high neutralizers the majority presented VNT 1:1280 and were equally distributed among 1:320, 1:640, and 1:2560 (**Supplementary Figure S1A**).

**Figure 1:**
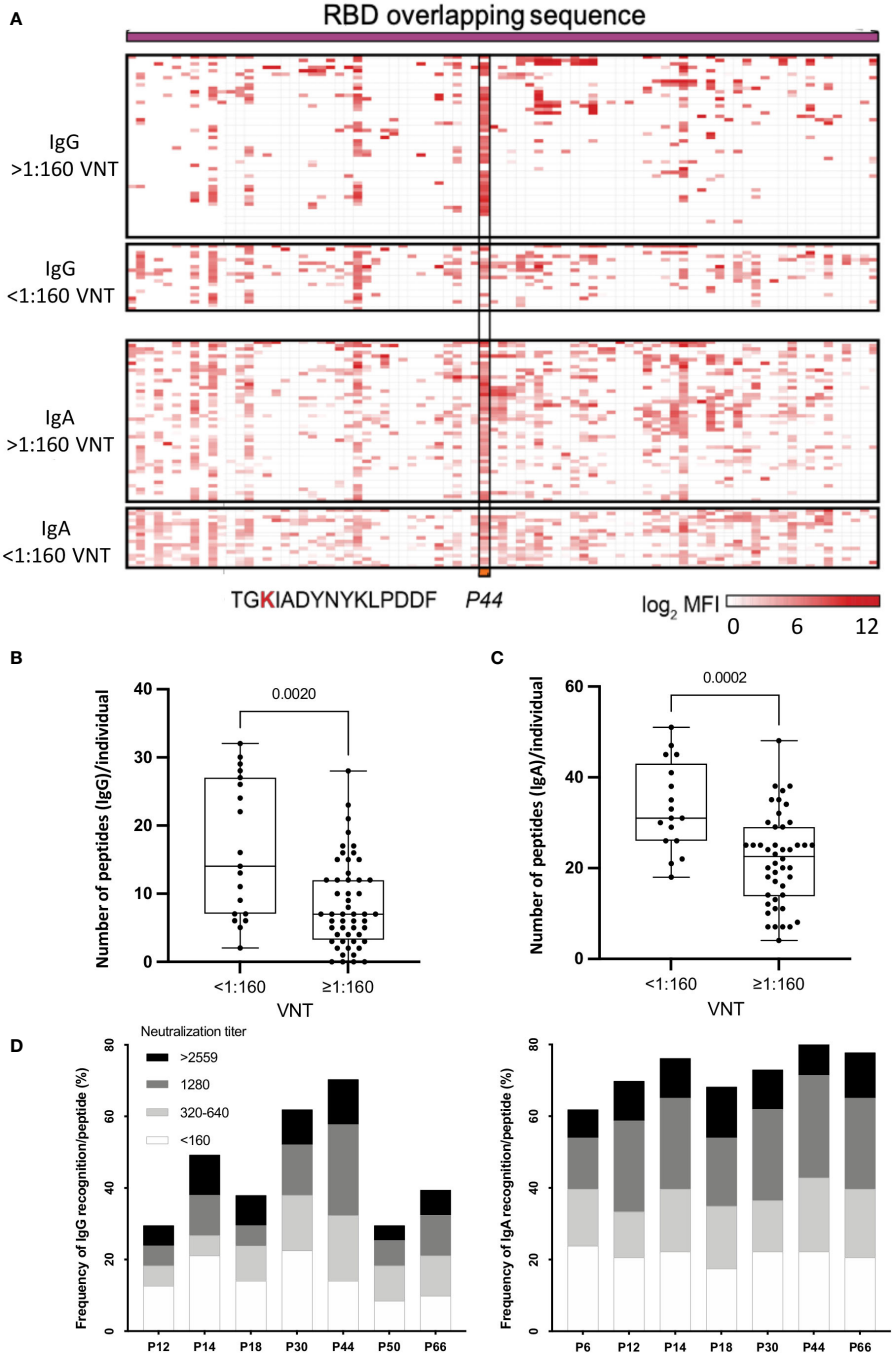
**(A)** Heatmap representing the magnitude of recognition of SARS-CoV-2 RBD peptides tested for IgG and IgA reactivity using a peptide microarray (columns: ordered by the primary structure sequence) for all convalescents (rows: grouped by antibody isotype and level of neutralizing activity tested in the same samples, and clustered using median values within each group). Highlighted is peptide *P44* (S_415-429_) and its respective amino acid sequence. High: individuals displaying serum with high neutralizing activity (≥1:160), Low: individuals displaying serum with low neutralizing activity (<1:160). MFI: mean fluorescence intensity. **(B)** Number of peptides recognized per individual considering high and low VNT for IgG and IgA **(C, D)** Selected immunodominant peptides recognized by IgG (*P12, P14, P18, P30, P44, P50* and *P66)* and IgA (*P6, P12, P14, P18, P30, P44* and *P66)*, of at least 30% of the individuals in the cohort (n=71) are represented with their total percentage of recognition and neutralization titers ranges. P6: peptide 6 (S_353-367_), P12: peptide 12 (S_365-379_), P14: peptide 14 (S_370-384_) and P18: peptide 18 (S_378-392_), P30: peptide 30 (S_397-401_), P44: peptide 44 (S_415-429_), P50: peptide 50 (S_427-441_) and P66: peptide 66 (S_459-473_).

We successfully identified linear anti-CoV-2 RBD antigenic regions for both IgG and IgA antibodies in the RBD microarray (**Figure 1A**). Overall, IgA recognition was broader, with 4 to 51 peptides being recognized per individual (median of 25 peptides), compared to IgG, with 0 to 32 peptides per individual (median of 7 peptides) (p <0.0001) (**Supplementary Figure S1B**).

Considering neutralization titers, we observed that individuals with VNT <1:160 recognized more peptides, for both IgG (**Figure 1B**) (p=0.002) and IgA (**Figure 1C**) (p=0.0002). On the other hand, individuals with higher VNT (≥1:160) recognized fewer peptides, indicating that high neutralization titers were associated with a less diverse peptide response. Next, we determined the topmost recognized peptides for both IgG and IgA reactivity. We detected eight peptides recognized by IgG and/or IgA (*P6, P12, P14, P18, P30, P44, P50* and *P66*) by at least 30% of individuals in the cohort. Of note, in contrast with IgG, for IgA antibodies, *P50* was not an immunodominant peptide, but *P6* was (**Figure 1D**).

Overall, individuals displaying IgG *P44*-reactivity presented mostly high VNT (>1:160) compared to individuals recognizing other peptides, while for IgA the VNT distribution was more homogeneous (**Figure 1D**).

### Immunodominant peptides are structurally localized within conserved regions among coronaviridae

Eight peptides (*P6, P12, P14, P18, P30, P44*, *P50* and *P66)* recognized by at least 30% of the convalescent cohort were considered immunodominant and therefore, selected for in-depth analysis.

**Figure 2:**
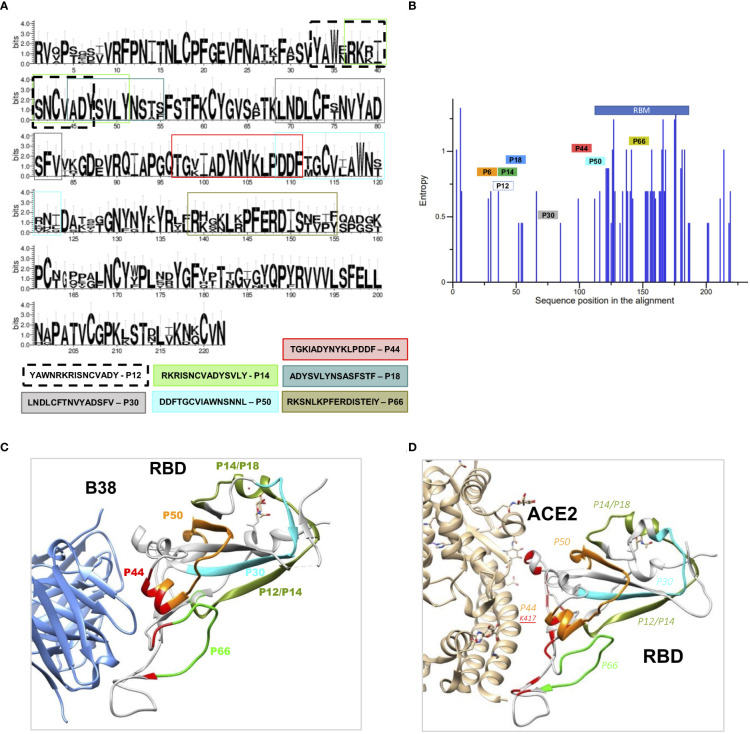
Structural and conservation evaluation of topmost recognized peptides **(A)** Conservation analysis of the top recognized peptides among RBD proteins from coronaviruses of the B lineage, SARS-CoV-2, SARS-CoV, SARS-SZ3, Rs3367-bat, CoV-pangolin, and RaTG13-bat. The height of the letters in the logo plot reflects the frequency of the amino acid in the multiple sequence alignment. **(B)** Entropy of aminoacid residues, top peptides highlighted, of the alignment made at **(A, C)**. RBD binding to the therapeutic neutralizing antibody B38 (pdb: 7BZ5) and with ACE-2 **(D)** interacting with P44, with contact amino acid residues in red. SARS-CoV-2 RBD (light gray). Colored regions show the most frequently recognized peptides identified among convalescents, namely, orange: P44: peptide 44 (S_415-429_), silver P6: peptide 6 (S_353-367_), golden P12, P14 and P18, peptide 12, 14, and 18 (S_365-379_, S_370-384_, S_378-392_), blue P30: peptide 30 (S_397-401_), violet P50, peptide 50 (S_427-441_) and green P66, peptide 66 (S_459-473_).

A conservation analysis of RBD proteins from coronaviruses of the B lineage, SARS-CoV-2, SARS-CoV, SARS-SZ3, Rs3367-bat, CoV-pangolin, and RaTG13-bat, showed that the selected peptides are located within well conserved regions (**Figure 2A**). The exceptions are P50 and P66 that lie within less conserved regions, although the positive charges of K and R amino acids seem to be conserved in these peptides (**Figure 2A**).

Therefore, we verified where these peptides are structurally located relative to virus binding sites of B38 (8), a therapeutic neutralizing antibody with binding sites around *P44* (**Figure 2B**) and ACE2 (**Figure 2C**). *P44* is the only peptide present in the RBD contact region with ACE2 (**Figure 2C**) and with the therapeutic nAb B38 (**Figure 2B**). In contrast to *P44*, peptides *P12, P14, P18* and *P30* are located outside the nAb B38 binding region (**Figure 2B**).

### Reactivity to the majority of immunodominant peptides does not correlate with VNT

**Figure 3:**
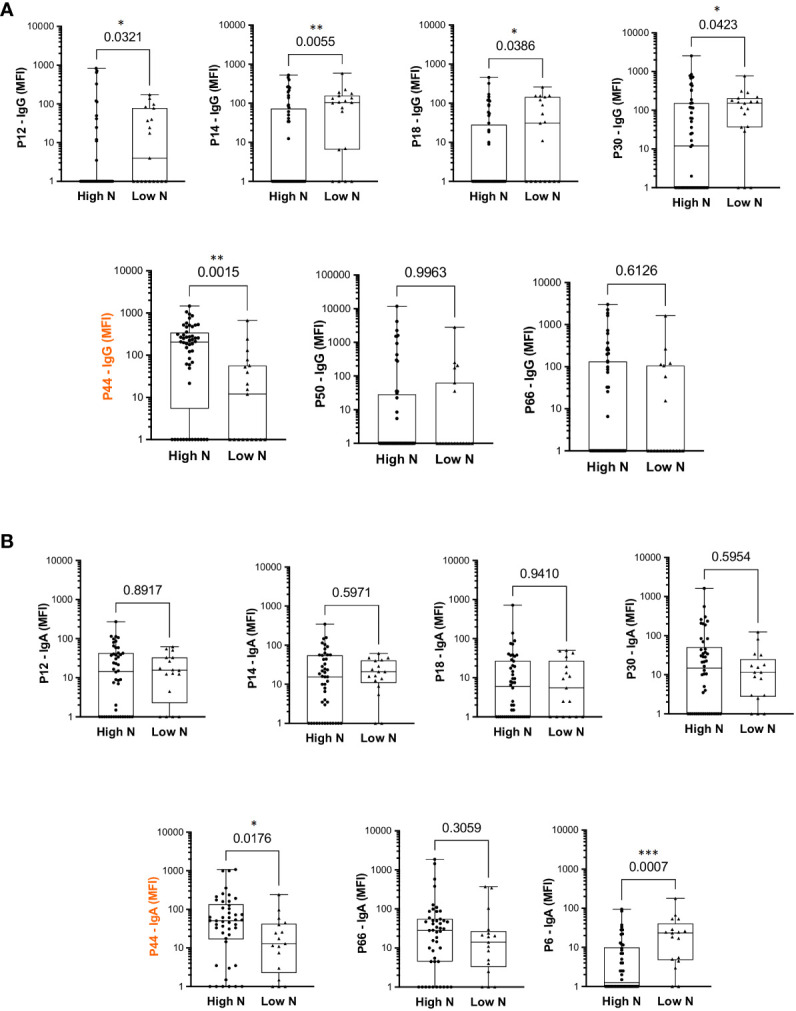
Differential intensity of reactivity to selected RBD peptides, comparing convalescents with high or low neutralizing capacity. **(A)** IgG profile. **(B)** IgA profile. High N: individuals with high neutralization capacity (≥1:160 virus neutralization titer); Low N: individuals with low neutralizing capacity (<1:160 virus neutralization titer); MFI: mean fluorescence intensity; the median is shown as a solid line, the box indicates the 25th and 75th percentiles, whiskers range from the highest to the lowest value (all data points shown). Mann Whitney *p<0.05; **p<0.001; ***p<0.0001.

The top eight peptides recognized by IgG and/or IgA (six recognized by both IgG and IgA) were analyzed considering relation to high or low serum Wuhan SARS-CoV-2 neutralizing capacity (**Figure 3**). *P44* was the only peptide significantly more recognized by high neutralizers (p=0.0015) for IgG and IgA (**Figure 3**). In contrast, *P12, P14, P18* and *P30* showed higher IgG binding intensities in individuals with low neutralization titers, as well as *P6* for IgA (**Figure 3**), suggesting being of lower relevance for virus neutralization. This is in line with the structural analyses showing that peptides *P12, P14, P18* and *P30* are located outside the nAb B38 binding region (**Figure 2B**). The intensity of IgG reactivity to *P50* and *P66* was not significantly different comparing high and low neutralizers (**Figure 3**).

### IgG and IgA reactivity to peptide P44 positively correlates with virus neutralization

**Figure 4:**
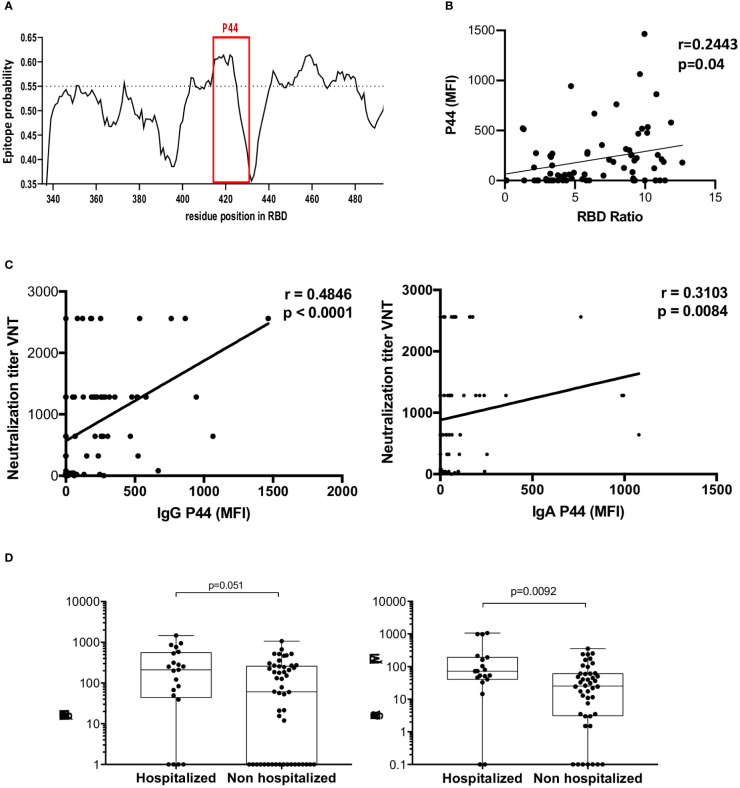
Profile of P44 recognition. **(A)** *In silico* epitope prediction using BepiPred. Highlighted is the region corresponding to P44, showing a high epitope predictive value. **(B)** Positive correlation of P44 MFI and RBD IgG recognition in ELISA. **(C)** Positive correlation of IgG and IgA MFI specific for P44 and virus neutralization titers (Spearman correlation for IgG r= 0.4846, p<0.0001, 95% confidence interval 0.2769 - 0.6491, and for IgA r=0.3103, p=0.0084, 95% confidence interval 0.07602 - 0.5121). **(D)** Mean fluorescence intensity (MFI) of P44 antibody recognition of hospitalized compared to non-hospitalized individuals, showing higher IgA reactivity in hospitalized individuals (p=0.0092, Mann Whitney).

Since *P44* (S_415-429_ TGKIADYNYKLPDDF) was the most recognized peptide, with 68% of convalescents showing IgG reactivity, and 82% IgA, and the only peptide more recognized by high neutralizers, we pursued further investigation. *In silico* B cell epitope predictions revealed that *P44* lies within an area with high epitope probability (**Figure 4A**). Noteworthy, *P44* IgG reactivity was positively correlated with neutralization titers (r= 0.4846, p<0.0001, 95% confidence interval 0.2769 - 0.6491). IgA reactivity also presented a weak but statistically significant correlation (r=0.3103, p=0.0084, 95% confidence interval 0.07602 - 0.5121) (**Figure 4C**). Notably, IgA reactivity to *P44* was higher in hospitalized individuals (p=0.0092), but the same did not occur for IgG (p=0.051) (**Figure 4D**). In addition, P44 signal intensities on the array also positively correlated (r=0.2443; p=0.04) with IgG levels for RBD measured in ELISA (**Figure 4B**).

Considering that *P44* bears the K417 mutation hotspot, we investigated the potential relevance of K417 mutations for antibody binding, by performing *in silico* equilibrium molecular dynamics simulations of beta (mutations: E484K, K417N, and N501Y) and gamma (mutations: E484K, K417T and N501Y) RBD variants interacting with the therapeutic mAb REGN10933, previously shown to present loss of neutralizing activity in a mutated K417N pseudovirus assay (18). Our structural results indicate that ancestral RBD presents several binding bridges nearby E484 residue, while gamma and beta variants do not (**Supplementary Figure S2**).

### P44 is located at the interface of neutralizing antibodies

**Figure 5:**
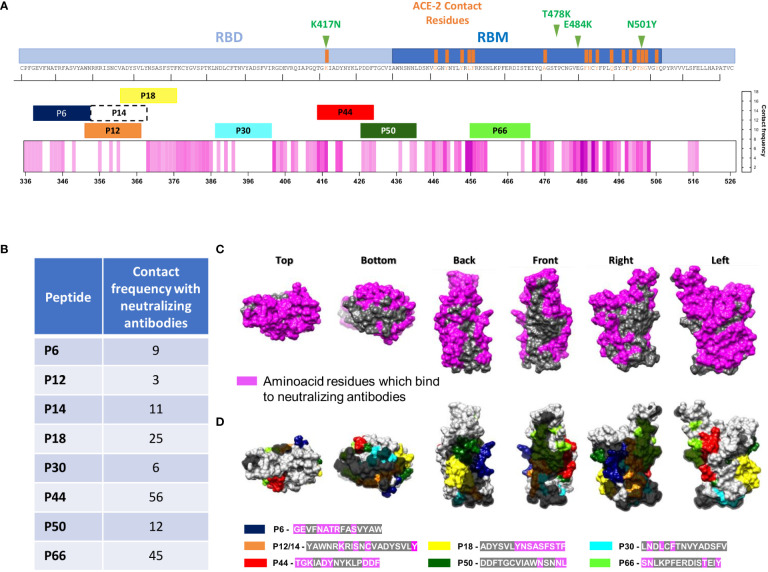
Structural localization of the eight most recognized peptides and monoclonal binding faces of RBD. **(A)** Linear RBD sequence showing the RBM region, highlighting ACE-2 contact residues. Top eight IgG and/or IgA recognized peptides (P6, P12, P14, P18, P30, P44, P50 and P66) are shown according to RBD residues. The pink heatmap represents the frequency at which nAbs contact each amino acid in RBD, according to a previous review (18). Mutation sites are shown in green in upper part. **(B)** Frequency of nAbs in contact with each amino acid in the RBD depicted in **(A, C)**. Surface structure of different RBD views showing regions where neutralizing antibodies bind to (in pink), and three non-neutralizing faces, in gray. **(D)** Top recognized peptides are shown in colors and non-neutralizing faces are highlighted in gray according to B.

Structural analysis suggested that *P44* lies within the recognition region of the nAb B38 (**Figure 2B**). We further investigated whether the other topmost peptides localized in regions already reported to be contact of nAbs, as shown in **Figure 5A**. Except for residue K417, all RBD residues that contact ACE-2 are located inside RBM (**Figure 5A**). *P44* displayed the highest contact frequency shared with nAbs (n=56), followed by P66 (n=45) (**Figure 5B**) although *P66* did not present statistically significant recognition by high neutralizers (**Figure 2**), nor correlation with neutralizing titers (data not shown). Structural analysis showed that RBD presents a large contact surface with nAbs, and three non-neutralizing interfaces that are not in contact with nAb target regions (18) (**Figure 5C**). By overlaying the amino acid sequences of the top peptides within these mapped binding areas to RBD surface, it is clearly observed that *P44* is almost entirely located within the binding regions of the nAbs. In fact, there are several interactions of *P44* with the nAbs, while the other peptides are mainly situated outside these regions (**Figure 5D**).

### Peptide recognition profile among vaccinated

Considering the great impact of vaccination for protection against SARS-CoV-2 infection and its potential role in the selection of novel variants, we examined RBD peptide antibody recognition profile in 18 individuals after 2 doses of the Coronavac vaccine and also after one booster of mRNA-1273 (Comirnaty).

**Figure 6:**
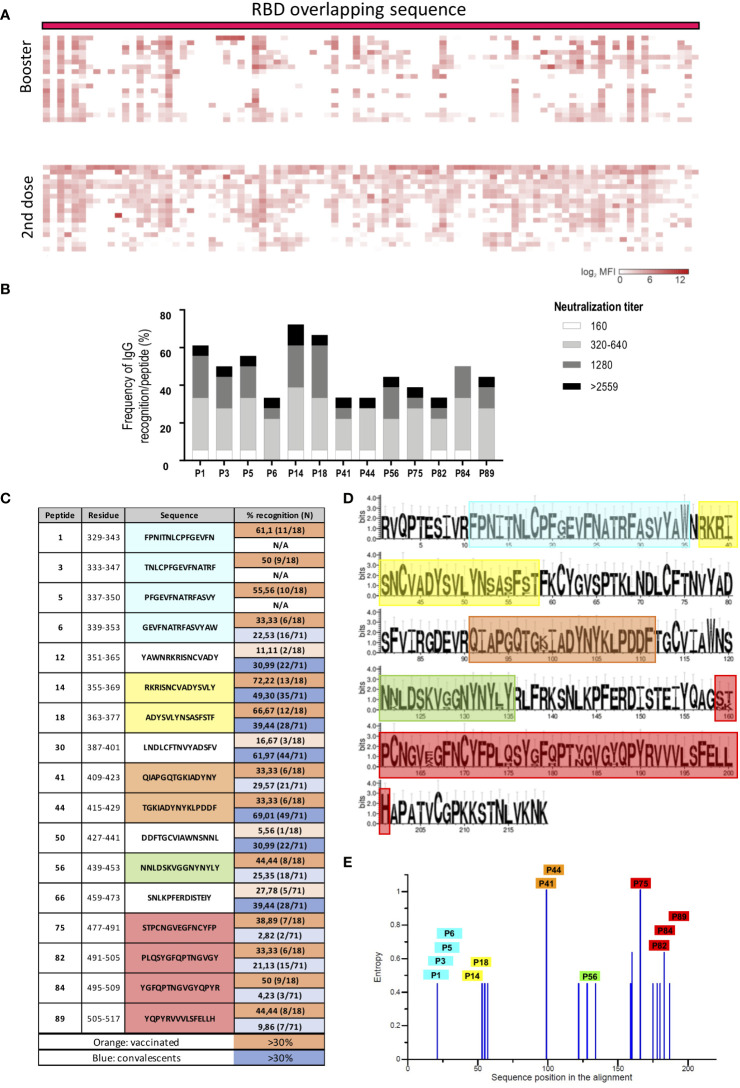
RBD peptide recognition profile of vaccinated individuals. **(A)** Heatmap representing the magnitude of recognition of SARS-CoV-2 RBD peptides tested for IgG reactivity using a peptide microarray (columns: ordered by the primary structure sequence) for all individuals (rows: grouped by vaccine regimen and clustered using median values within each group). 2^nd^ dose: individuals who received two doses of Coronavac. Booster: individuals who received two doses of Coronavac followed by one dose of MiRNA-273 (Comirnaty). **(B)** Selected immunodominant peptides recognized by IgG by at least 30% of the individuals in the vaccinated cohort (n=18), showing the total percentage of recognition and neutralization titer ranges. **(C)** List of top peptides among convalescents or vaccinated. **(D)** Conservation analysis of the top recognized peptides among RBD proteins from different VOCs (alfa, beta, gamma, delta and omicron BA.1) compared to Wuhan. The height of the letters in the logo plot reflects the frequency of the amino acid in the multiple sequence alignment. **(E)** Entropy of amino acid residues considering the alignment performed, as in D with top peptides highlighted.

We found 13 peptides that were recognized by more than 30% of the cohort, showing a more diverse repertoire than convalescents (**Figures 6A, B**). From those, only *P14, P18* and *P44* are coincident with top peptides recognized by convalescents (**Figure 6C**). A conservation analysis comparing Wuhan with alpha, beta, gamma, delta and omicron BA.1 VOCs showed that these peptides lie within less conserved regions (**Figure 6D**), or regions presenting high degree of entropy (**Figure 6E**).

**Figure 7:**
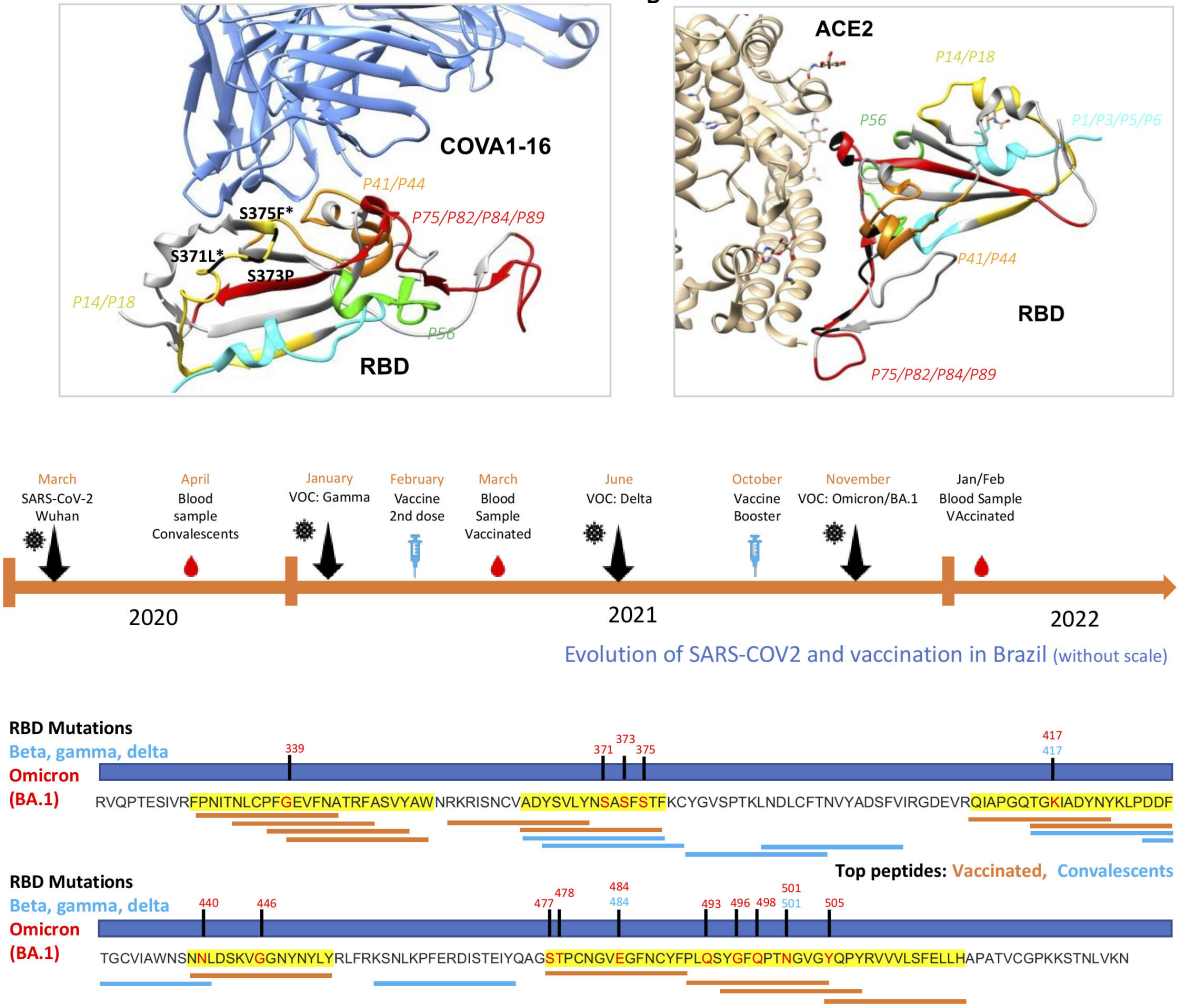
Structural evaluation of topmost recognized peptides among vaccinated and analysis of occurrence at mutation sites. **(A)** RBD binding to the therapeutic neutralizing antibody COVA1-16 (pdb:7JMW) and with ACE-2 **(B)** interacting with P14/18, the most recognized region, with contact amino acid residues in black. SARS-CoV-2 RBD (light gray). Colored regions show the most frequently recognized peptides identified among vaccinated. **(C)** Timeline showing collection period of samples, vaccination and surge of VOCs in Brazil. **(D)** Linear RBD sequence showing mutation sites of Beta, Gamma, Delta and Omicron BA.1. Regions highlighted in yellow are the most recognized among vaccinated. Top thirteen from vaccinated (orange) and top seven (blue) IgG recognized peptides are shown according to RBD residues.

Next, we verified where these peptides are structurally located relative to virus binding sites to COVA1-16, a therapeutic neutralizing antibody with binding sites in the region of peptides *P14* and *P18*, the most recognized region among vaccinated (**Figure 7A**) and ACE2 (**Figure 7B**). Contact residues with COVA1-16, S371 and S375, are mutated in Omicron BA.1, S3171L and S375F (**Figure 7A**). Besides *P44*, we found *P75, P82, P84 and P89* located in the RBD contact region with ACE2 (**Figure 7B**).

Since the immunodominant RBD epitope profiles of IgG recognition were distinct between the convalescent and vaccinated groups, we raised the hypothesis of differential immune pressure taking place along the vaccination period, in comparison with the surge of VOCs, in Brazil (**Figure 7C**). Interestingly, these novel regions, dominantly recognized among vaccinated, are located in regions that are mutated in Omicron BA.1, an important variant emerging after massive vaccination (**Figure 7D**).

## Discussion

The identification of B cell epitopes that induce the production of neutralizing antibodies is critical to understand the dynamics of virus evolution. In addition, it also provides relevant information to improve strategies for the development of vaccines against SARS-CoV-2 and its continuously emerging new variants. Our peptide array approach provided a comprehensive profile of IgG and IgA reactivity to Wuhan SARS-CoV-2 derived peptides, in convalescents from the first wave of the pandemic as well as in vaccinated individuals without previous infection, allowing us to identify a set of mostly different immunodominant peptides. Reactivity to the topmost immunodominant peptide (*P44*) amongst convalescents positively correlated with high neutralizing capacity, suggesting that this region is an important neutralization target. Further, we detected that this topmost immunodominant peptide comprises a hotspot mutation site reported for several variants, as Gamma, Delta and Omicron. Peptide recognition repertoire differed from convalescents to vaccinated. Since we did not evaluate individuals with hybrid immunization, SARS-Cov-2 antigenic stimulation - vaccine and infection – we cannot specify which repertoire of peptides would be the most recognized by both exposures.

Overall, for convalescents, IgA peptide recognition profiles were more diverse than IgG profiles, different from what was found in a cohort of COVID-19 convalescents using Virscan, showing that IgG and IgA recognize the same protein regions with similar frequencies across the population viral (19). Notably, a more constrained repertoire of IgG-recognized peptides was found in convalescents associated with higher neutralization titers. If, on one hand, a more antigen restricted Ab response can indicate a more focused and robust immune response, on the other, it could also favor the selection of escape variants. In line with this interpretation, it was recently reported that, due to humoral immune imprinting, Omicron breakthrough infections caused significant reductions in the epitope diversity of NAbs and increased proportion of non-neutralizing mAbs. This in turn would have concentrated humoral immune pressure and promoted convergent evolution (20).

Peptides recognized by more than 30% of our cohort were defined as immunodominant (21). We first determined the reactivity profile of convalescents and only antibody reactivity to the topmost immunodominant peptide, *P44* (S_415-429_ TGKIADYNYKLPDDF), positively correlated with neutralizing capacity, suggesting that responses to the other immunodominant peptides may not have, individually, generated antibodies with neutralizing capacity.

*P44* encompasses the mutation hotspot residue K417 present in the gamma (K417T), beta and omicron (K417N) VOCs, which have been shown to be less recognized by neutralizing antibodies from both Covid-19 first wave convalescents (21) and anti-SARS-CoV-2 vaccinated individuals (22). This residue contacts the human ACE-2 and is likely a result of adaptive evolution of the SARS-CoV-2, displaying stronger receptor binding (23). Selective pressure could have favored new variants’ immune evasion from a potent established humoral response directed to this residue, possibly due to robust humoral response against this immunodominant region. Recognition of other immunodominant peptides was not correlated with neutralizing activity and these peptides are not within hotspot mutation sites.

It has been shown that broadly neutralizing monoclonal antibodies that recognize RBD epitopes that are conserved among SARS-CoV-2 variants and other sarbecovirus outside the receptor-binding motif were able to neutralize Omicron (24). Several monoclonal antibodies cross react with and neutralize sarbecoviruses beyond the SARS-CoV-2 clade, indicating that neutralizing antibodies targeting the sarbecovirus conserved region remain effective (24, 25).

Conformational data from the literature indicate that mutations occurring at residues with higher structure-based antibody accessibility scores, such as residue 417 when RBD is in the open form, are more likely to comprise antibody recognition epitopes (26). Accordingly, our data show that amino acid substitutions in this region can impact the polyclonal antibody response.

The very same *P44* sequence has been shown to be target of IgG antibodies from some COVID-19 convalescents and recipients of Spike-mRNA vaccines (27). Although neutralizing capacity was not evaluated in these studies and very few individuals were analyzed, these results support the immunodominant nature of this peptide, herein also observed for IgA. Intriguingly, in two microarray studies performed with smaller peptides of 12-mer overlapping the *P44* sequence, no antibody reactivity directed to T^415^GKIADYNYKLP^426^ was found (3, 28). This could be due to the short length of the peptide used in that study and suggest we have been able to detect the precise epitope, within our peptide. This is also corroborated by the fact we did not find a dominant antibody reactivity directed to the flanking peptides of *P44* (*P43* and *P45*), but in fact we found the opposite, very low recognition. On the other hand, sera from COVID-19 convalescent individuals reacted with longer peptides containing the *P44* sequence, such as the peptides G^413^QTGIADYNYKLPDDFTGC^432^ (29) and V^407^RQIAPGQTGIADYNYKLPDDFTGCVIAW^436^ (5), reinforcing our data.

Furthermore, we should mention *that P44* localizes close to the binding sites of several therapeutic antibodies, frequently shown to overlap with the ACE2 binding site^20^. In fact, the therapeutic neutralizing antibodies REGN10933 (7) and B38 (8) recognize the core RBD binding region containing *P44* sequence. Our data show that *P44* presents 56% of contact frequency with neutralizing antibodies. Also, others have shown that all sites at which mutations strongly escape binding are in direct (<4 Å) or proximal (4–8 Å) contact with antibodies in resolved structures (9). Accordingly, the exchange of the positively charged K417 to the neutral asparagine (beta variant) or threonine (gamma variant) has been shown to promote a shift in the helix structure (30), further increasing the distance of molecular interaction, possibly impairing antibody binding. The half-maximal inhibitory concentration (IC50) of REGN10933 dropped 13.0- and 8.2-fold against beta and gamma, respectively, largely because of the K417N/T and E484K mutations (31). Therefore, antibodies recognizing the observed sequential peptide within the *P44* region would possibly fail to neutralize variants with K417N mutation, as previously reported (30–32). Of note, residue E484, another hotspot mutation site, is in a molecular loop stabilized by two close disulfide bonds (26), probably establishing a conformational but not a linear antibody binding epitope, probably explaining why reactivity to this peptide was not detected in our peptide array.

Therefore, we raise that immunodominant epitopes may favor immune pressure and the selection of variants bearing hotspot mutations, capable of evading antibody response. To put forward this hypothesis we analyzed the peptide profile of individuals vaccinated and not previously infected. It is important to highlight that the variety of peptides being most recognized among vaccinated was greater than among convalescents. Also, the number of RBD mutations occurring in Omicron are higher compared to previous VOCs (alpha, beta, delta and gamma). The emergence of the same mutation sites occurring independently in different parts of the globe may reflect the adaptation of SARS-CoV-2 to humans against a background of increasing immunity (33).

Our results showed that amongst convalescents a more focused response, with fewer peptides being recognized, was associated with higher neutralization titers. We reason that immune pressure following vaccination contributed to epitope spreading and likely surge of omicron that presents several mutations at RBD and the capacity of escaping antibody neutralization.

An important challenging issue is that all current vaccines express the ancestral SARS-CoV-2 Spike, whereas currently circulating variants such as Omicron have several mutations that promote evasion of the immune response. The so-called “original antigenic sin”, that describes the phenomenon in which the development of immunity against pathogens/antigens is shaped by the first exposure to a related pathogen/antigen (34), could be an additional hurdle to developing broadly neutralizing vaccines based on mutated spike. The virus continues to adapt in humans, and further divergence from the initial Wuhan sequences is expected. Accordingly, such vaccines could, in fact, boost responses to conserved epitopes rather than induce responses to the new variants. It is uncertain if a broad immunity conferred by a bivalent vaccine containing Wuhan and Omicron strains will prove to be the best choice (35) especially considering the antigenic sin phenomenon that may impair specific responses to Omicron (36). Maybe the next steps will be to develop specific vaccines based on novel dominant and/or subdominant variants without boosting the index strain, as shown by promising results in pre-clinical assays (37).

In summary, we have identified a set of antibody immunodominant sequential peptides contained in Wuhan SARS-CoV-2 RBD that differs from convalescents of the first wave of infection with Wuhan strain and vaccinated individuals following two or three-dose regimen. Among convalescents, only reactivity to the most frequently recognized peptide, *P44*, positively correlated with neutralization titers, suggesting that it comprises neutralizing antibody epitopes due to its RBD localization. This immunodominant B-cell RBD peptide harbors a leading mutation hotspot site in three independent SARS-CoV-2 VOCs: beta, gamma, and omicron. Further, RBD immunodominant peptides are more diverse among vaccinated and were detected in regions that coincide with mutation sites of Omicron BA.1, a very relevant VOC emerging after massive vaccination worldwide. Our results provide evidence that immunodominant epitopes recognized by convalescent and mainly by vaccinated may favor selective pressure for variants bearing mutations at these sites, favoring immunological escape.

## COVID-19 SP-Brazil Team in alphabetical order

Aline M. A. Martins, André Kenji Honda, Andreia Cristina Kazue Kuramoto Takara, Ariane Cesario Lima, Cesar Sato, Deibs Barbosa, Fernanda Romano Bruno, Edgar Ruz Fernandes, Giuliana Xavier De Medeiros, Greyce Luri Sasahara, João Paulo Silva Nunes, Juliana de Souza Apostolico Andrade, Lucas Caue Jacintho, Luis Antonio Rodrigues Carneiro, Marco Antonio Stephano, Marcos Camargo Knirsch, Marcio Massao Yamamoto, Maria Lucia Carnevale Marin, Mirian Pinheiro Bruni, Rafael Ribeiro Almeida, Raquel Elaine de Alencar, Ricardo José Giordano, Roberta Liberato Pagni, Samar Freschi de Barros, Sandra Maria Monteiro, Simone Regina dos Santos.

## Data availability statement

The raw data supporting the conclusions of this article will be made available by the authors, without undue reservation.

## Ethics statement

The studies involving human participants were reviewed and approved by CONEP (Comissão Nacional de Ética em Pesquisa) (CAAE: 30155220.3.0000.0068). The patients/participants provided their written informed consent to participate in this study.

## Author contributions

JO and KS: Data curation, Investigation, visualization, Formal Analysis, and Writing - original draft. JK, GG, VC, KS, EC-N, SB, DR, and ED: Data curation and Investigation. JO: Writing - review & editing. CR, GG, HA, and DS: Conceptualization, Formal Analysis, Investigation, Visualization, Methodology, Software, Writing - review & editing. MF, CR, and HN: Formal Analysis, Investigation, Methodology, Software, Writing - review & editing. RM, JM, ID, AU, GS, and RS: Visualization, Methodology and Writing - review & editing. KS, EC-N, and JK: Conceptualization, Funding acquisition, Project administration, Writing - original draft, Writing - review & editing. All authors contributed to the article and approved the submitted version.
