# Supplementary material for: Immunodominant antibody responses directed to SARS-CoV-2 hotspot mutation sites and risk of immune escape
Source: Front Immunol. 2023 Jan 5;13:1010105. doi: 10.3389/fimmu.2022.1010105 (PMC9849925; doi:10.3389/fimmu.2022.1010105)
Supplement: Supplementary file 1 [file DataSheet_1.docx]

Supplementary Material

**Supplementary Table S1 –** Peptides immobilized in microarray.

| **Peptide #** | **Amino acid sequence** | **Position at Spike SARS-CoV-2 from Ancestral Wuhan Hu-1 isolate** |
| --- | --- | --- |
| P1 | GSGSGSGCPFGEVFN | 335-342 |
| P2 | GSGSGCPFGEVFNAT | 335-344 |
| P3 | GSGCPFGEVFNATRF | 335-346 |
| P4 | GCPFGEVFNATRFAS | 335-348 |
| P5 | PFGEVFNATRFASVY | 337-350 |
| P6 | GEVFNATRFASVYAW | 339-353 |
| P7 | VFNATRFASVYAWNR | 341-355 |
| P8 | NATRFASVYAWNRKR | 343-357 |
| P9 | TRFASVYAWNRKRIS | 345-359 |
| P10 | FASVYAWNRKRISNC | 347-361 |
| P11 | SVYAWNRKRISNCVA | 349-363 |
| P12 | YAWNRKRISNCVADY | 351-365 |
| P13 | WNRKRISNCVADYSV | 353-367 |
| P14 | RKRISNCVADYSVLY | 355-369 |
| P15 | RISNCVADYSVLYNS | 357-371 |
| P16 | SNCVADYSVLYNSAS | 359-373 |
| P17 | CVADYSVLYNSASFS | 361-375 |
| P18 | ADYSVLYNSASFSTF | 363-377 |
| P19 | YSVLYNSASFSTFKC | 365-379 |
| P20 | VLYNSASFSTFKCYG | 367-381 |
| P21 | YNSASFSTFKCYGVS | 369-383 |
| P22 | SASFSTFKCYGVSPT | 371-385 |
| P23 | SFSTFKCYGVSPTKL | 373-387 |
| P24 | STFKCYGVSPTKLND | 375-389 |
| P25 | FKCYGVSPTKLNDLC | 377-391 |
| P26 | CYGVSPTKLNDLCFT | 379-393 |
| P27 | GVSPTKLNDLCFTNV | 381-395 |
| P28 | SPTKLNDLCFTNVYA | 383-397 |
| P29 | TKLNDLCFTNVYADS | 385-399 |
| P30 | LNDLCFTNVYADSFV | 387-401 |
| P31 | DLCFTNVYADSFVIR | 389-403 |
| P32 | CFTNVYADSFVIRGD | 391-405 |
| P33 | TNVYADSFVIRGDEV | 393-407 |
| P34 | VYADSFVIRGDEVRQ | 395-409 |
| P35 | ADSFVIRGDEVRQIA | 397-411 |
| P36 | SFVIRGDEVRQIAPG | 399-413 |
| P37 | VIRGDEVRQIAPGQT | 401-415 |
| P38 | RGDEVRQIAPGQTGK | 403-417 |
| P39 | DEVRQIAPGQTG**K**IA | 405-419 |
| P40 | VRQIAPGQTG**K**IADY | 407-421 |
| P41 | QIAPGQTG**K**IADYNY | 409-423 |
| P42 | APGQTG**K**IADYNYKL | 411-425 |
| P43 | GQTG**K**IADYNYKLPD | 413-427 |
| P44 | TG**K**IADYNYKLPDDF | 415-429 |
| P45 | **K**IADYNYKLPDDFTG | 417-431 |
| P46 | ADYNYKLPDDFTGCV | 419-433 |
| P47 | YNYKLPDDFTGCVIA | 421-435 |
| P48 | YKLPDDFTGCVIAWN | 423-437 |
| P49 | LPDDFTGCVIAWNSN | 425-439 |
| P50 | DDFTGCVIAWNSNNL | 427-441 |
| P51 | FTGCVIAWNSNNLDS | 429-443 |
| P52 | GCVIAWNSNNLDSKV | 431-445 |
| P53 | VIAWNSNNLDSKVGG | 433-447 |
| P54 | AWNSNNLDSKVGGNY | 435-449 |
| P55 | NSNNLDSKVGGNYNY | 437-451 |
| P56 | NNLDSKVGGNYNYLY | 439-453 |
| P57 | LDSKVGGNYNYLYRL | 441-455 |
| P58 | SKVGGNYNYLYRLFR | 443-457 |
| P59 | VGGNYNYLYRLFRKS | 445-459 |
| P60 | GNYNYLYRLFRKSNL | 447-461 |
| P61 | YNYLYRLFRKSNLKP | 449-463 |
| P62 | YLYRLFRKSNLKPFE | 451-465 |
| P63 | YRLFRKSNLKPFERD | 453-467 |
| P64 | LFRKSNLKPFERDIS | 455-469 |
| P65 | RKSNLKPFERDISTE | 457-471 |
| P66 | SNLKPFERDISTEIY | 459-473 |
| P67 | LKPFERDISTEIYQA | 461-475 |
| P68 | PFERDISTEIYQAGS | 463-477 |
| P69 | ERDISTEIYQAGSTP | 465-479 |
| P70 | DISTEIYQAGSTPCN | 467-481 |
| P71 | STEIYQAGSTPCNGV | 469-483 |
| P72 | EIYQAGSTPCNGVEG | 471-485 |
| P73 | YQAGSTPCNGVEGFN | 473-487 |
| P74 | AGSTPCNGVEGFNCY | 475-489 |
| P75 | STPCNGVEGFNCYFP | 477-491 |
| P76 | PCNGVEGFNCYFPLQ | 479-493 |
| P77 | NGVEGFNCYFPLQSY | 481-495 |
| P78 | VEGFNCYFPLQSYGF | 483-497 |
| P79 | GFNCYFPLQSYGFQP | 485-499 |
| P80 | NCYFPLQSYGFQPTN | 487-501 |
| P81 | YFPLQSYGFQPTNGV | 489-503 |
| P82 | PLQSYGFQPTNGVGY | 491-505 |
| P83 | QSYGFQPTNGVGYQP | 493-507 |
| P84 | YGFQPTNGVGYQPYR | 495-509 |
| P85 | FQPTNGVGYQPYRVV | 497-511 |
| P86 | PTNGVGYQPYRVVVL | 499-513 |
| P87 | NGVGYQPYRVVVLSF | 501-515 |
| P88 | VGYQPYRVVVLSFEG | 503-517 |
| P89 | YQPYRVVVLSFEGSG | 505-517 |
| P90 | PYRVVVLSFEGSGSG | 507-517 |
| P91 | RVVVLSFEGSGSGSG | 509-517 |
| **color code** | | |
| orange | strong reactivity with only IgG | |
| blue | strong reactivity with only IgA | |
| violet | strong reactivity with both IgG and IgA | |


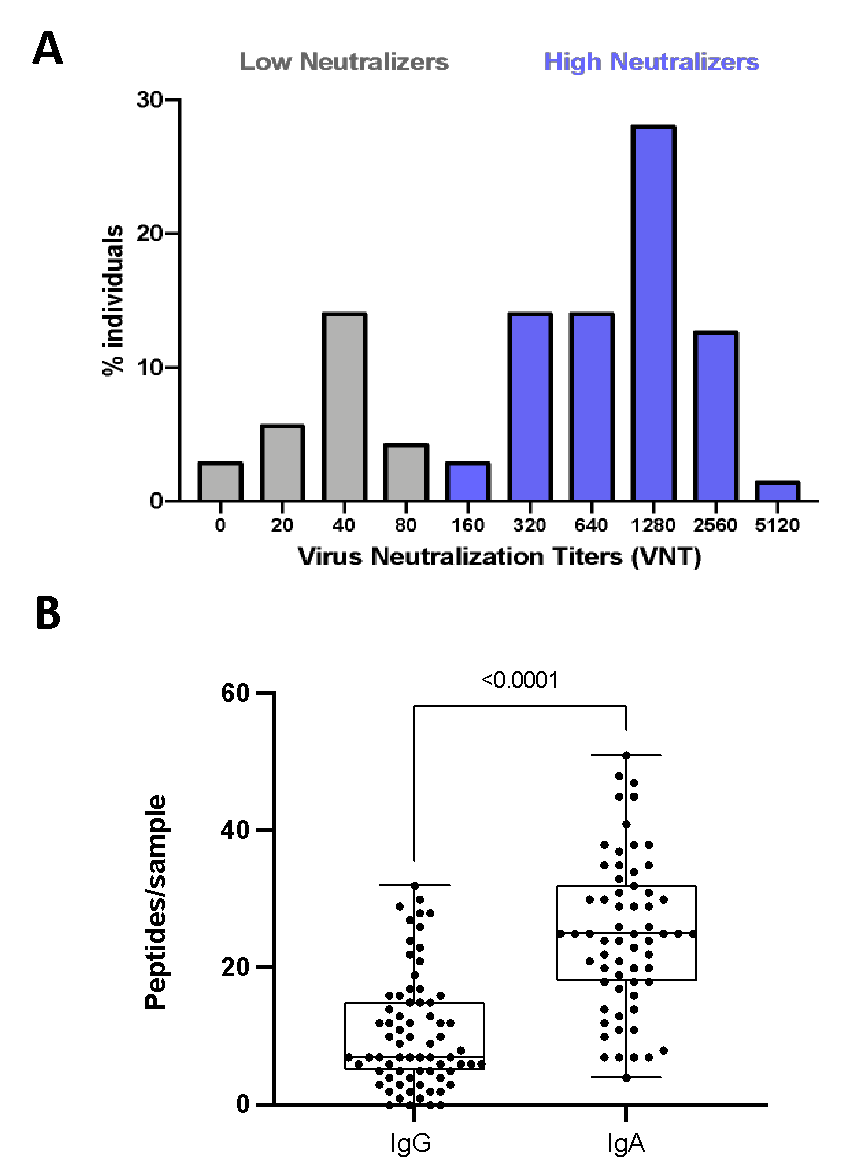


**Supplementary Figure S1. A.** Percentage of individuals according to virus neutralizing capacity (n=71). **B**. Number of peptides being recognized per patient’s serum. MFI=Mean Fluorescence Intensity.

**Supplementary Figure S2.** Structure of RBD binding to monoclonal antibody, REGN10933 (pdb: 6XDG) showing ancestral RBD without mutations (K417, E484 and N501), Beta that contains mutations K417N, E484K and N501Y and gamma carrying mutations K417T, E484K and N501Y after docking simulation using Dockthor tool. **A.** Structure bonds showing Ancestral RBD presents several binding bridges nearby residue E484 while gamma and beta do not. **B.** Surface solvent accessibility evidencing charged regions. In blue, positive charges and in red negative charges. Highlighted is the region of contact between mAb and RBD.
